# Supplementary material for: Design, Optimization, Manufacture and Characterization of Milbemycin Oxime Nanoemulsions
Source: Pharmaceutics. 2025 Feb 22;17(3):289. doi: 10.3390/pharmaceutics17030289 (PMC11944943; doi:10.3390/pharmaceutics17030289)
Supplement: Supplementary file 1 [file pharmaceutics-17-00289-s001.zip › pharmaceutics-3449616-supplementary.pdf]

Supplementary Materials

Design, Optimization, Manufacture and Characterization of Milbemycin Oxime Nanoemulsions

Ze-En Li, Yang-Guang Jin, Shao-Zu Hu, Yue Liu, Ming-Hui Duan, Shi-Hao Li, Long-Ji Sun, Fan Yang * and Fang Yang *

College of Animal Science and Technology, Henan University of Science and Technology, Luoyang 471023, China

* Correspondence: Fan Yang: fyang@haust.edu.cn or yfscau@126.com; Fang Yang: yfhaust@126.com.

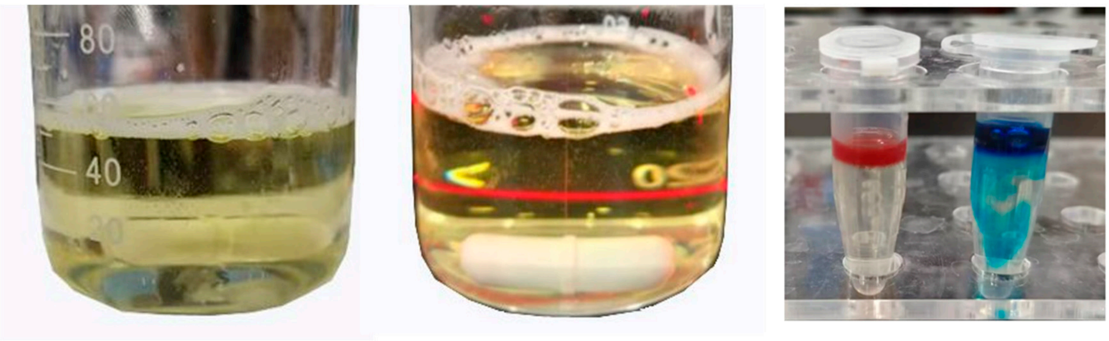

(a) (b) (c)

Figures S1. Figure S1**a** shows the appearance of milbemycin oxime nanoemulsions; Figure S1**b** shows the Tyndall effect of the nanoemulsions; and Figure S1**c** shows the type identification of the nanoemulsions.

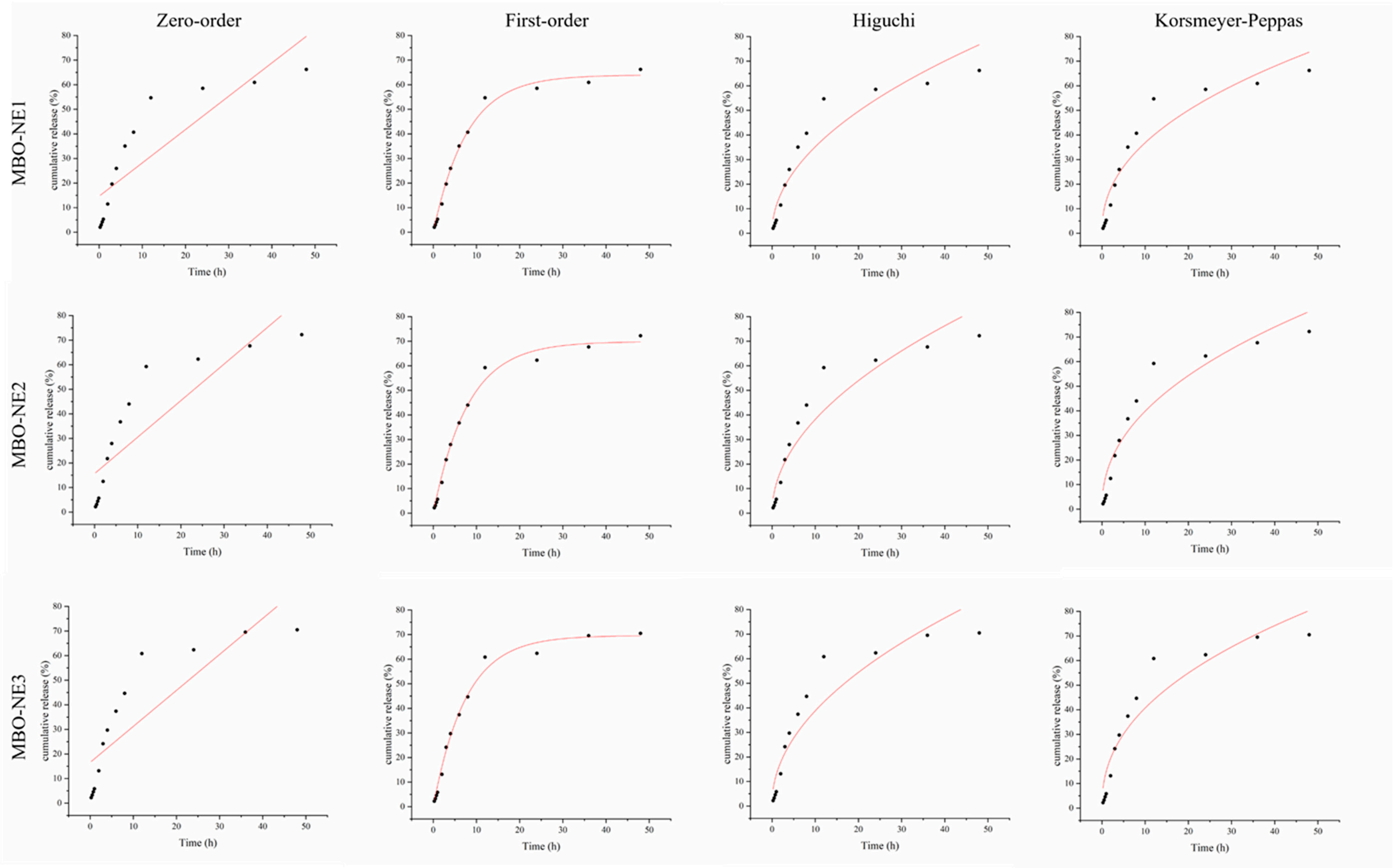

Figures S2. The *in vitro* release curves fitting for the three milbemycin oxime nanoemulsions (MBO-NE1, MBO-NE2, and MBO-NE3) prepared under identical conditions.

The vertical axis displays the three replicates of milbemycin oxime nanoemulsions (MBO-NE1, MBO-NE2, and MBO-NE3).

The horizontal axis in the fitting analysis represents the different kinetic models used to fit the release data for milbemycin oxime nanoemulsions. From left to right, these models are Zero-order, First-order, Higuchi, and Korsmeyer-Peppas models.
